# How air pollution fuels respiratory infections in children: current insights

**DOI:** 10.3389/fpubh.2025.1567206

**Published:** 2025-04-28

**Authors:** Susanna Esposito, Valentina Fainardi, Annachiara Titolo, Angela Lazzara, Marialaura Menzella, Beatrice Campana, Alberto Argentiero, Nicola Principi

**Affiliations:** ^1^Department of Medicine and Surgery, Pediatric Clinic, University Hospital, University of Parma, Parma, Italy; ^2^Professor Emeritus, University of Milan, Milan, Italy

**Keywords:** air pollution, air quality improvement, respiratory infections, particulate matter, children’s health, prenatal exposure

## Abstract

**Background:**

Air pollution is a significant global health concern, particularly for younger children who are especially susceptible to its adverse effects. Pollutants such as particulate matter (PM), nitrogen oxides (NO and NO₂), sulfur dioxide (SO_2_), ozone (O_3_), and carbon monoxide (CO) are associated with increased risks of upper respiratory tract infections (URTI) and lower respiratory tract infections (LRTI). While this association is well-documented, there are critical gaps in understanding the magnitude of these risks, the roles of specific pollutants, and the influence of age, sex, and exposure duration.

**Methods:**

To confirm the relationship between air pollution and respiratory tract infections in children and to identify areas for further research on reducing pollution-related respiratory damage, a literature review was conducted using the MEDLINE/PubMed database for studies published from January 2000 to December 2024. Eligible studies included randomized controlled trials, cohort studies, and meta-analyses focusing on the relationship between air pollution and respiratory infections in children. Studies were grouped by pollutant type, exposure timing, and infection type.

**Results:**

The literature analysis confirmed that pollution significantly increases the risk of URTI and LRTI in children, with infants and young children being the most vulnerable. Potential mechanisms for the development of respiratory tract pollution-related diseases include the promotion of oxidative stress, induction of inflammatory responses, deregulation of the immune system, and genetic alterations. Prenatal exposure significantly alters respiratory tract development, increasing the risk of LRTI and acute otitis media (AOM) early in life. Both short-term and long-term postnatal exposures can cause severe and recurrent LRTIs, reducing quality of life and leading to frequent hospitalizations and early death. However, the available data do not allow for precise definition of the magnitude of the risk, the individual and combined roles of specific pollutants, and the influence of factors such as age, sex, duration, and site of exposure on the development and severity of respiratory infections. Inconsistent findings on pollutant combinations and specific diseases like otitis media highlight the need for further research.

**Conclusion:**

Air pollution is a major risk factor for respiratory infections in children, both prenatal and postnatal exposure can have significant negative impact. However, present knowledge is inadequate to develop effective preventive and therapeutic measures. Further studies are needed to minimize these cultural limits. In particular, it is necessary to delve deeper into how the various pollutants circulate, how they interact with each other, and how they are influenced by climate change and other environmental drivers. Results of these key researches can be translate into clinical and public health practice capable to help protect and improve children’s environmental health.

## 1 Background

Air pollution, as defined by the World Health Organization (WHO), refers to the alteration of the natural characteristics of the atmosphere by any chemical, physical, or biological agent contaminating the indoor or outdoor environment. It is recognized as one of the leading causes of morbidity and mortality worldwide (1). The health impacts of air pollution are profound, contributing to an increased risk of cardiovascular and respiratory diseases, lung cancer, and strokes. In 2021, air pollution was responsible for 8.1 million deaths globally, a figure that is projected to rise given the inadequate mitigation of climate change, which exacerbates the issue (2). Of these pollution-related deaths, 1.7 million occurred in children under the age of 5, underscoring their heightened vulnerability to the adverse health effects of ambient air pollution (3).

In children, the clinical impact of air pollution differs from that in adults. While cardiovascular manifestations predominate in adults (accounting for over 60% of air pollution-related deaths) children are more prone to respiratory conditions (4). However, exposure to pollutants in children has also been associated with severe neuropsychiatric outcomes (5). Several biological and behavioral factors contribute to this increased susceptibility. The developing respiratory system in children is highly sensitive to environmental insults, with air pollution interfering with both structural and functional growth as early as the fetal stage (6), potentially causing persistent and irreversible bronchial and pulmonary damage (7). Additionally, children breathe more rapidly than adults, inhaling greater volumes of air relative to their body size (8), and they often engage in outdoor activities during periods of poor air quality (9). Compounding these factors is their immature immune system, which makes them more susceptible to respiratory infections, further increasing their vulnerability to air pollution (10).

Reduced lung function, the development of asthma, and an elevated risk of respiratory infections are among the most common respiratory conditions associated with air pollution in children. Prospective studies have demonstrated a strong link between ambient air pollution and significant lung function deficits, with improvements in air quality shown to partially reverse these effects (11–17). Regarding asthma, it has long been established that short-term exposure to air pollution exacerbates symptoms in children already diagnosed with the condition (18). More recent studies have provided compelling evidence that long-term exposure to pollutants, particularly particulate matter (PM) and nitrogen oxides (NO and NO₂), may contribute to asthma development in previously healthy children (19, 20).

However, the relationship between air pollution and the risk of respiratory infections remains less well-defined. Despite the Global Burden of Disease Study has included upper respiratory infections (URTIs) and lower respiratory tract infections (LRTIs) among diseases attributable to ambient air pollution (21), the association is still debated, with limited definitive data on the roles of specific pollutants or their combinations. Furthermore, key variables such as duration of exposure, sex, and age remain inadequately explored. These gaps hinder the development of evidence-based policies and interventions to improve child health outcomes. Additionally, the vicious cycle of air pollution and infections—where infections amplify the negative effects of pollution and pollution exacerbates infection risk—remains unbroken. The primary aim of this study is to synthesize current knowledge on the potential link between air pollution and respiratory infections in children, to identify areas of controversy and contested claims, and to highlight any gaps that may exist in research to date. Identifying unresolved questions in this area may pave the way for more effective strategies to mitigate these risks and enhance child health.

## 2 Methods

To achieve the study’s objective, a comprehensive literature review was conducted using the MEDLINE/PubMed database, covering studies published between January 2000 and December 2024. The review prioritized high-quality evidence, including randomized placebo-controlled trials, controlled clinical trials, double-blind randomized controlled studies, systematic reviews, and meta-analyses. Articles were included if they were published in English, involved subjects of pediatric age, and examined the relationship between air pollution and respiratory infections. Exclusion criteria encompassed non-English language publications, studies with insufficient or incomplete data, non-peer-reviewed articles, duplicates, unavailable full texts, or abstract-only papers.

The search strategy used combinations of keywords such as “Air Pollution” OR “Particulate matter” OR “Nitric Oxides” OR Carbon Oxide” OR!Air Quality Improvement” AND “Respiratory Infection” OR “Upper respiratory tract infections” OR “Otitis Media” OR “Lower respiratory tract infections” OR “Pneumonia” AND “Child” OR “Adolescent.”

All studies identified through the database search were screened for relevance by two independent reviewers, VF and NP, based on their titles and abstracts. For studies that appeared to meet the inclusion criteria, or when relevance could not be definitively determined from the title or abstract alone, the full text was obtained for further assessment. The final decision regarding inclusion was made after a detailed evaluation of the full text against the predefined criteria. Any disagreements between VF and NP were resolved through consultation with a third independent reviewer, SE.

The data extracted from the included studies were grouped and analyzed according to their relevance in defining the impact of various air pollutants on respiratory infections. Specifically, the analysis focused on upper respiratory tract infections, including otitis media, and lower respiratory tract infections, such as pneumonia. The findings were synthesized to provide a comprehensive overview of the relationships between different types of air pollutants and the risk of respiratory infections in children.

## 3 Results

### 3.1 Air pollutants

Table 1 shows the key air pollutants and their sources. Particulate matter (PM), nitrogen oxides (NO and NO₂), ground-level ozone (O₃), sulfur dioxide (SO₂), and carbon monoxide (CO) are among the air pollutants with the most significant adverse health impacts (21). PM is a complex mixture of solid particles and liquid droplets, categorized based on aerodynamic equivalent diameter into coarse particles (PM₁₀), fine particles (PM₂.₅), and ultrafine particles (UFP). PM₁₀ includes particles with diameters of 10 μm or smaller, PM₂.₅ includes particles 2.5 μm or smaller, while UFP consists of nanoscale particles. Table 1 lists major air pollutants and their sources. Significant pollutants with severe health effects include PM, NO and NO₂, ground-level ozone (O₃), sulfur dioxide (SO₂), and carbon monoxide (CO) (22). PM consists of solid and liquid particles, categorized by size: coarse (PM₁₀, ≤ 10 μm), fine (PM₂.₅, ≤ 2.5 μm), and ultrafine particles (UFPs, <0.1 μm or 100 nm). UFPs are also called nanoparticles (NPs), nanoaerosols (NAs), and PM0.1. NPs refer to engineering materials released into the environment, “UFP” is common in toxicological studies, and “PM0.1” is used in atmospheric pollution research (23, 24).

**Table 1:** Summary of key air pollutants and their sources.

Air pollutant	Description	Primary sources
PM10	Particles ≤ 10 μm in diameter; primarily affect upper airways	Construction, dust, industrial activities
PM2.5	Particles ≤ 2.5 μm in diameter; can penetrate alveoli	Vehicle emissions, wildfires, industrial processes
UFP	Ultrafine particles; nanoscale size, highly toxic	Combustion engines, industrial emissions
NO	Reactive gas formed during combustion; precursor to NO2	Combustion engines, industrial emissions
NO2	Nitrogen dioxide; formed from NO reaction with O3	Combustion processes, vehicle emissions
O3	Ozone; secondary pollutant formed from VOC and NO2 reactions	Industrial emissions, vehicle exhaust, VOC reactions
SO2	Sulfur dioxide; primarily from fossil fuel combustion	Coal and oil combustion, industrial emissions
CO	Carbon monoxide; formed by incomplete combustion of fuels	Incomplete combustion of fossil fuels, wood burning

PM originates from various sources, including fuel combustion, industrial activities, wildfires, wood burning, gravel pits, agricultural operations, and dusty roads (primary PM) (25). Common components of PM include sodium chloride, elemental carbon, trace metals, and minerals. Additionally, PM can form from chemical reactions involving gasses such as SO₂, NO, and NO₂, reacting with organic compounds from both natural and human-made sources (secondary PM) (26).

Inhalation of PM varies depending on particle size. PM₁₀ primarily impacts the upper airways due to its larger diameter, whereas smaller particles, such as PM₂.₅, can penetrate deeper into the lungs and reach the alveoli (22, 27). Ultrafine particles can descend even further, increasing the potential for respiratory and systemic damage. Moreover, the composition of PM also influences its toxicity. Ultrafine particles containing transition metals and organic species, despite contributing minimally to the total mass of PM, exhibit high intrinsic toxicity. These findings prompted the WHO in 2021 to recommend lowering the annual air quality guideline level for PM₂.₅ from 10 μg/m^3^ to 5 μg/m^3^ (27).

Gaseous pollutants primarily result from combustion processes, particularly fossil fuel combustion (28). For example, NO reacts with O₃ to produce NO₂, while ground-level O₃ forms through interactions between NO₂ and volatile organic compounds (VOCs) emitted from human activities. VOCs include substances such as ammonia, amines, aldehydes, hydrogen sulfide, and volatile hydrocarbons (28).

Although criteria for defining air pollution vary between countries depending on local characteristics, target values based on average exposure indicators for each pollutant are commonly established, particularly in industrialized regions. Additionally, several institutions provide guidelines for ambient air pollution levels that are considered tolerable. These guidelines serve as reference tools for air quality management and policy planning (29, 30).

Air quality is often assessed through the Air Quality Index (AQI), a standardized scale derived from measurements of air concentrations of major pollutants. The AQI ranges from 0 to 500, with higher values indicating worse air quality. An AQI below 50 is considered safe, whereas values above 100 are deemed unhealthy, particularly for sensitive populations (31).

### 3.2 Disease mechanisms

The impact of air pollution on respiratory tract damage and the risk of respiratory infections is influenced by several factors, including site of deposition, penetration capacity, bioavailability, and the long residence time of pollutants in the air (Table 2). While each air pollutant may exhibit specific toxic properties, most damage leading to increased susceptibility to respiratory infections stems from shared mechanisms. These include the promotion of oxidative stress, induction of inflammatory responses, deregulation of the immune system, and genetic alterations (32, 33). However, much of the available data originates from *in vitro* studies, and it remains unclear whether the concentrations of pollutants causing tissue damage in experimental settings are representative of real-world exposures in humans (34).

**Table 2:** Evidence linking air pollutants to respiratory infections.

Pollutant	Main references	Respiratory outcome	Key findings
PM2.5	(70, 71)	Increased risk of LRTI, impaired lung development	Prenatal exposure linked to LRTI risk (RR = 1.34, 95% CI 1.09–1.66)
PM10	(85)	URTI and LRTI in children, worsened asthma symptoms	Short-term exposure increases risk of URTI by 10%
NO2	(87–89)	Strongly associated with URTI, inflammation, immune modulation	NO2 exposure strongly correlated with pediatric URTI
O3	(89)	Increased inflammatory biomarkers, exacerbates URTI	10 μg/m^3^ increase associated with 1.05% rise in inflammation markers
SO2	(60, 86, 90)	Linked to pneumonia risk, inflammatory pathways activation	10 μg/m^3^ exposure increases pneumonia hospitalization by 10.44%

#### 3.2.1 Oxidative stress

Oxidative stress results from an excessive production of reactive oxygen species (ROS), including oxygen radicals, hydroxyl radicals, and highly reactive forms of oxygen such as hydrogen peroxide (H₂O₂) and singlet oxygen (O₂) (22). Under normal physiological conditions, ROS play essential roles in modulating cellular processes, such as growth factor signaling, hypoxic responses, inflammation, and immune regulation (35). These beneficial effects occur only when ROS levels are tightly controlled. Excessive ROS production can lead to the oxidation of DNA, proteins, and lipids, resulting in genomic instability, protein dysfunction, and cellular damage or death (36, 37). Lipid peroxidation further exacerbates cell dysfunction (38).

To counteract these effects, cells maintain antioxidant networks composed of enzymes like superoxide dismutase (SOD), catalase, and glutathione peroxidase (GPx), along with low-molecular-weight scavengers such as glutathione (GSH), uric acid, and coenzyme Q (39). These antioxidants ensure ROS levels remain in balance, preserving their physiological benefits (40). Unfortunately, many air pollutants, including ultrafine particles (UFP), PM₂.₅, ozone (O₃), nitrogen oxides (NO and NO₂), and transition metals, are potent oxidants or stimulate ROS production (41). When pollutant levels rise, ROS production exceeds the neutralizing capacity of antioxidants, leading to oxidative stress and subsequent tissue damage (42).

In the respiratory tract, oxidative stress from air pollution causes mucus hypersecretion, damage to lung macrophages and bronchial epithelial cells, inactivation of antiproteases, bronchial wall edema, and bronchoconstriction (43–46).

#### 3.2.2 Inflammatory response, immune system deregulation, and genetic changes

Oxidative stress and direct exposure to air pollutants trigger severe inflammation and dysregulation of antimicrobial and antiviral immunity in the respiratory tract, increasing the risk of infections (47). Studies have primarily focused on PM and NO₂. A systematic review of 55 studies found that *in vitro* exposure of bronchial epithelial cells and macrophages to PM₂.₅ (50–100 μg/mL for 9–24 h) significantly increased the production of pro-inflammatory cytokines, including IL-1α, IL-1β, IL-6, IL-8, and GM-CSF (48). This promotes lung injury while reducing interferon-β (IFN-β) secretion, a key antiviral cytokine (49). PM also activates the NLRP3 inflammasome in epithelial cells, producing bioactive IL-1β and amplifying inflammation (47). Furthermore, PM reduces alveolar macrophage motility, impairs mucociliary clearance, inhibits bacterial phagocytosis (50, 51), and promotes airway pathogen growth when containing iron (52).

PM exposure also enhances dendritic cell maturation, leading to increased Th2/Th17 cytokine levels and decreased Th1 cytokine expression. This shift induces inflammation and promotes infiltration of inflammatory cells into respiratory tissues (53). Some of these effects may be mediated by PM-induced epigenetic modifications, such as DNA methylation, histone modifications, and microRNA regulation. For instance, PM₂.₅ exposure has been associated with methylation of key inflammation-regulating genes, including IFN-γ, IL-4, IL-10, and Foxp3, resulting in lower Treg cell levels (54–56).

NO₂ has similar effects on inflammation and immunity. Exposure to NO₂ at levels common in areas with heavy traffic or in households with gas stoves increases the release of GM-CSF, IL-8, and TNF-α from airway epithelial cells (57). Repeated exposure also induces Th2-mediated cytokine production, including IL-5, IL-10, and IL-13, suggesting a pro-allergic effect (58). Additionally, NO₂ increases ICAM-1 expression in epithelial cells, a major receptor for rhinoviruses and respiratory syncytial viruses, explaining its role as a risk factor for recurrent respiratory infections (58). NO₂ exposure during pregnancy has also been linked to DNA methylation of mitochondria-related genes in offspring and altered expression of antioxidant defense genes (59).

Although less extensively studied, other gaseous pollutants like O₃ and SO₂ exhibit similar inflammatory and immune-modulating effects. Xu et al. reported that a 10 μg/m^3^ increase in short-term exposure to O₃ and SO₂ resulted in significant increases of 1.05% (95% confidence interval [CI] 0.09–2.02) and 10.44% (95% CI 4.20–17.05), respectively, in C-reactive protein levels, a major inflammatory biomarker (60).

### 3.3 Clinical evidence of the impact of air pollutants on the risk of respiratory infections

The relationship between air pollution and the susceptibility of children to respiratory infections is well-documented through numerous studies. Increased incidences of both upper respiratory tract infections (URTIs) and lower respiratory tract infections (LRTIs) have been observed across all pediatric age groups, with infants, toddlers, and preschool children showing the highest vulnerability. This association is evident following both short- and long-term exposure to air pollution. Notably, prenatal exposure to pollutants has also been linked to increased respiratory infection rates after birth (61–65). The following sections summarize key findings that highlight the effects of air pollution on respiratory health.

#### 3.3.1 Prenatal exposure

Air pollutants, particularly PM, can cross the placenta and enter the fetal circulatory system, with detectable amounts found in fetal tissues as early as the first and second trimesters of pregnancy (66, 67). These pollutants can disrupt normal lung development, increasing susceptibility to infections through mechanisms such as oxidative stress, impaired placental function, and epigenetic modifications (68). Given that lung organogenesis begins shortly after conception and continues throughout pregnancy and postnatal life (69), early exposure can lead to significant structural and functional alterations, predisposing the lungs to infections.

Evidence suggests that earlier exposure during pregnancy correlates with more severe lung damage. For instance, the effects of prenatal PM₁₀ exposure are more pronounced in preterm infants compared to full-term newborns, indicating that underdeveloped lungs are particularly vulnerable (70). Additionally, transient tachypnea and respiratory distress syndrome are more prevalent in infants with prenatal exposure to pollutants such as O₃, CO, PM₂.₅, and PM₁₀ (71). Long-term studies reveal that lung damage caused by prenatal exposure persists, with children showing impaired lung function up to 9 years of age (72–74).

The link between prenatal exposure and infection susceptibility has also been highlighted. A cohort study involving infants hospitalized for respiratory problems found that intrauterine exposure to high PM₂.₅ levels (>24 μg/m^3^) during the first and second trimesters increased the risk of LRTI (1st trimester, relative risk [RR] 1.31, 95% CI 1.08–1.60; 2nd trimester, RR = 1.34, CI 95% 1.09–1.66) (75). A separate study reported that maternal exposure to industrial pollutants such as PM₁₀ and SO₂ significantly raised the risk of pneumonia in their offspring (odds ratio [OR] 1.83, 95% CI 1.59–2.11 for PM₁₀ and OR 3.43, 95% CI 2.83–4.17 for SO₂) (76).

PM₂.₅ exposure during pregnancy has been specifically linked to increased LRTI risk in children during their first year of life, with higher third-trimester exposure showing a significant association (RR = 1.06, 95% CI 1.00–1.13) (77). Furthermore, prenatal exposure to air pollutants such as PM₂.₅, SO₂, and NO₂ has been associated with an increased risk of acute otitis media (AOM) in childhood, with ORs of 1.43 (95% CI 1.19–1.71), 1.18 (95% CI 1.01–1.37), and 1.18 (95% CI 1.00–1.39), respectively (78).

#### 3.3.2 Short-term exposure in infants and children

The link between transient exposure to air pollution and the development of URTIs and LRTIs has been studied extensively worldwide. Most studies show that even brief exposure to pollutants significantly increases the risk of respiratory infections (79–84).

##### 3.3.2.1 Upper respiratory tract infections

A Polish study involving 1,475 children aged 3–12 years found that higher PM₂.₅ and PM₁₀ concentrations over a 12-week period were associated with a 10% increase in URTI incidence in areas with the highest pollution levels compared to those with the lowest (85). Similarly, a Chinese study on daily air pollution and outpatient visits for URTI in children aged 0–14 years reported a positive association between pollutant levels and infection risk. For a 10 μg/m^3^ increase in PM₂.₅, PM₁₀, SO₂, NO₂, and CO concentrations, the excess risk (ER) of URTI was 0.15% (95% CI 0.07–0.23), 0.38% (95% CI 0.17–0.60), 2.92% (95% CI 1.88–3.97), 4.47% (95% CI 3.69–5.25), and 0.05% (95% CI 0.02–0.08), respectively (86). NO₂ appeared to have the strongest effect, as confirmed by previous studies (87, 88).

##### 3.3.2.2 Lower respiratory tract infections

A meta-analysis of 17 studies demonstrated that short-term exposure to PM₂.₅, PM₁₀, SO₂, O₃, and NO₂ increased the risk of pneumonia, with ER per 10 μg/m^3^ increase in PM₂.₅ and PM₁₀ being 1.8% (95% CI 0.5–3.1) and 1.5% (95% CI 0.6–2.4), respectively (89). In a U.S. study, each 10 μg/m^3^ increase in PM₂.₅ was associated with a 15–32% rise in healthcare encounters for LRTI, with the greatest effect seen after three weeks of exposure (90).

A Korean study of 713,588 children hospitalized for LRTI found that a 10 μg/m^3^ increase in the 7-day moving average of PM₂.₅ concentrations increased hospital admissions by 1.20% (95% CI 0.71–1.71) in children aged 0–5 years (84). Seasonal variations were noted, with stronger effects during the warm season.

#### 3.3.3 Long-term exposure to air pollution

Long-term exposure to air pollution is strongly linked to increased respiratory infections, as evidenced by higher child mortality rates due to LRTI in regions with poor air quality. The 2019 Global Burden of Disease (GBD) Study reported 691,373 deaths in children under five attributable to PM exposure, with the vast majority occurring in low- and middle-income countries (91). Reducing household air pollution has been shown to lower pneumonia incidence significantly, emphasizing the benefits of long-term air quality improvements (92).

The ESCAPE project, involving 10 European birth cohorts, found that long-term exposure to PM₁₀ and NO₂ increased pneumonia risk in children under 2 years, with ORs of 1.76 (95% CI 1.00–3.09) and 1.30 (95% CI 1.02–1.65) per 10 μg/m^3^ increase, respectively (93). Additionally, PM₂.₅ exposure during early childhood was linked to AOM risk, with the highest impact seen in the first year of life (94).

Further evidence comes from a study by Hertz-Picciotto et al., which reported a 30% increased risk of bronchitis in children under two for every 25 μg/m^3^ increase in PM₂.₅ exposure over a 30-day average (RR 1.30, 95% CI 1.08–1.58) (64). Similarly, Lanari et al. confirmed the association between PM₂.₅ and hospitalization for RSV bronchiolitis in children, particularly those living near highways (95, 96).

## 4 Discussion

The available evidence clearly demonstrates that air pollution poses a significant threat to children’s respiratory health, with a well-established link to an increased risk of both URTIs and LRTIs. However, despite the undeniable association between air pollution and respiratory infections, several critical aspects remain unresolved. These include the precise magnitude of the risk, the individual and combined roles of specific pollutants, and the influence of factors such as age, sex, duration, and site of exposure on the development and severity of respiratory infections.

A major challenge in addressing these questions is the significant heterogeneity across studies. Variability in study populations, methodologies for defining and measuring pollution, types and combinations of pollutants examined, exposure durations, outcome assessments, and analytical approaches have led to conflicting results. Furthermore, many studies suffer from methodological limitations, such as inadequate control for confounding factors and reliance on indirect measures of exposure. Additionally, the majority of research has been conducted in high-income or upper-middle-income countries, where pollution levels and population characteristics differ from those in low-income countries, limiting the generalizability of the findings.

Prenatal exposure to air pollution and its association with subsequent LRTI risk illustrates these challenges. A recent systematic review by Pepper et al. (97), which analyzed 16 publications covering 12 research studies, highlights the difficulties in drawing reliable conclusions. While the review suggested a potential positive association between prenatal PM₂.₅ exposure and increased LRTI risk, the overall evidence was inconclusive. The small number of studies, high risk of bias in exposure and outcome assessments, and lack of data from low-income settings underscore the need for further research in this area.

Similar issues arise in studies of postnatal exposure to air pollution. As highlighted in this review, the true impact of individual air pollutants and their combinations remains inadequately defined. Most studies evaluate pollutants in isolation, overlooking the potential synergistic effects of multiple pollutants. For example, the study by Lu et al. demonstrates this limitation; while all common pollutants appeared to increase URTI risk when assessed individually, only NO₂ remained significantly associated when full-pollutant models were applied. This finding underscores the complexity of interactions between pollutants and the need for more comprehensive analyses.

The impact of air pollution on specific respiratory diseases also requires further clarification. While the association between air pollution and pneumonia appears robust, its role in promoting conditions such AOM and bronchiolitis is less clear, with studies producing conflicting results. Additionally, the mechanisms by which individual pollutants exert their harmful effects remain incompletely understood. For instance, PM appears to affect respiratory tissues in ways that go beyond its mass, with evidence suggesting that the chemical composition of PM may play a critical role in tissue damage. Unfortunately, studies investigating PM composition and its impact on health are scarce, highlighting another important gap in the current knowledge.

## 5 Conclusion

Air pollution remains a major and persistent risk factor for respiratory infections in children, contributing to a significant burden of disease and mortality. Both prenatal and postnatal exposure to pollutants such as PM, nitrogen oxides (NO and NO₂), SO₂, O₃, and CO have been linked to increased susceptibility to respiratory infections, impairing lung development and compromising immune defenses (98–100). Despite extensive evidence supporting these associations, substantial knowledge gaps remain, limiting the development of targeted and effective public health interventions.

Future research is urgently needed to elucidate the specific mechanisms through which air pollution exacerbates respiratory infections (Table 3). Understanding the cellular and molecular pathways by which pollutants induce oxidative stress, inflammation, immune dysregulation, and epigenetic modifications in pediatric populations is crucial. Investigating the individual and combined effects of various pollutants, including emerging contaminants such as UFP and VOCs, will help clarify their synergistic or antagonistic interactions. Long-term cohort studies in diverse geographical and socio-economic settings are necessary to better assess chronic exposure risks, regional variations in pollution levels, and health disparities among different populations.

**Table 3:** Gaps in knowledge and research needs.

Research gap	Description
Combined pollutant effects	Limited studies on how pollutants interact to exacerbate health risks
Prenatal vs. postnatal exposure impacts	Differences in vulnerability based on exposure timing remain unclear
Regional variations in air pollution and health outcomes	Data from low-income regions with high pollution levels are scarce
Pollutant-specific mechanisms of damage	Mechanisms such as epigenetic changes and immune modulation are not fully understood
Longitudinal studies on chronic outcomes	Need for long-term studies to evaluate chronic respiratory outcomes

Differentiating the impact of air pollution exposure at various stages of lung development, from *in utero* to early childhood, will identify critical windows of susceptibility and potential interventions. Additionally, examining how climate change influences pollutant dispersion, seasonal variations, and their interactions with respiratory infections, particularly in vulnerable pediatric populations, will provide a more comprehensive understanding of the issue. Assessing the effectiveness of public health interventions, including air quality regulations, indoor air purification, behavioral modifications, and novel pharmacological or nutritional strategies, is essential for mitigating pollution-related damage.

While further research is essential, existing mitigation strategies should be reinforced to provide immediate protection. Stricter air quality standards, enhanced pollution monitoring, promotion of clean energy sources, and community-based interventions—such as reducing indoor exposure to biomass fuel combustion—are critical steps in reducing the burden of air pollution on children’s respiratory health. Additionally, integrating environmental health considerations into pediatric healthcare policies can improve early diagnosis and management of pollution-related respiratory conditions.

Addressing these knowledge gaps and implementing evidence-based policies will be crucial in developing more targeted, effective, and sustainable approaches to protecting children from the harmful effects of air pollution. By fostering interdisciplinary collaboration among environmental scientists, healthcare providers, and policymakers, we can advance our understanding of air pollution’s health impact and pave the way for innovative solutions that enhance children’s well-being worldwide.
